# A high-throughput screening to identify small molecules that suppress *huntingtin* promoter activity or activate *huntingtin-antisense* promoter activity

**DOI:** 10.1038/s41598-021-85279-2

**Published:** 2021-03-17

**Authors:** Houda G. Khaled, Hongxuan Feng, Xin Hu, Xin Sun, Wang Zheng, Pan P. Li, Dobrila D. Rudnicki, Wenjuan Ye, Yu-Chi Chen, Noel Southall, Juan Marugan, Christopher A. Ross, Marc Ferrer, Mark J. Henderson, Russell L. Margolis

**Affiliations:** 1 Laboratory of Genetic Neurobiology, Division of Neurobiology, Department of Psychiatry, Johns Hopkins University School of Medicine, CMSC 8-121, 600 N. Wolfe Street, Baltimore, MD 21287 USA; 2 National Center for Advancing Translational Sciences, NIH, Rockville, MD USA; 3 Departments of Neuroscience and Pharmacology, Johns Hopkins University School of Medicine, Baltimore, MD USA; 4 Department of Neurology, Johns Hopkins University School of Medicine, Baltimore, MD USA; 5 Present Address: Center for Neural Science, New York University, New York, NY USA; 6 Present Address: Jacobio Pharmaceuticals Ltd., Beijing, China; 7 Present Address: Center for Cancer and Immunology Research, Children’s National Medical Center, Washington, DC 20010 USA

**Keywords:** Drug development, Huntington's disease, High-throughput screening, High-throughput screening, Huntington's disease, High-throughput screening, Gene expression, Gene regulation, Stem cells, Embryonic stem cells, Long non-coding RNAs, Non-coding RNAs, Transcriptional regulatory elements

## Abstract

Huntington’s disease (HD) is a neurodegenerative disorder caused by a CAG repeat expansion in exon 1 of *huntingtin *(*HTT*). While there are currently no disease-modifying treatments for HD, recent efforts have focused on the development of nucleotide-based therapeutics to lower HTT expression. As an alternative to siRNA or oligonucleotide methods, we hypothesized that suppression of HTT expression might be accomplished by small molecules that either (1) directly decrease HTT expression by suppressing *HTT* promoter activity or (2) indirectly decrease HTT expression by increasing the promoter activity of *HTT-AS,* the gene antisense to *HTT* that appears to inhibit expression of *HTT*. We developed and employed a high-throughput screen for modifiers of *HTT* and *HTT-AS* promoter activity using luminescent reporter HEK293 cells; of the 52,041 compounds tested, we identified 898 replicable hits. We used a rigorous stepwise approach to assess compound toxicity and the capacity of the compounds to specifically lower huntingtin protein in 5 different cell lines, including HEK293 cells, HD lymphoblastoid cells, mouse primary neurons, HD iPSCs differentiated into cortical-like neurons, and HD hESCs. We found no compounds which were able to lower huntingtin without lowering cell viability in all assays, though the potential efficacy of a few compounds at non-toxic doses could not be excluded. Our results suggest that more specific targets may facilitate a small molecule approach to HTT suppression.

## Introduction

Huntington’s disease (HD) is an autosomal dominant neurodegenerative disorder characterized by abnormalities of movement, cognition, and emotion, with relentless progression to death^1^. HD is caused by expansion of a CAG repeat in exon 1 of the ubiquitously expressed *Huntingtin *(*HTT*) gene; the repeat is in-frame to encode polyglutamine^2^. In individuals with HD, the mutant huntingtin protein (mHTT) containing a long polyglutamine expansion is found throughout the CNS and also in non-CNS tissues^3^. HD pathogenesis is thought to derive primarily from a toxic gain-of-function conferred on mHTT by the polyglutamine expansion^4^, although some evidence exists for other forms of toxicity, including loss-of-function^5,6^ and toxicity from the mutant mRNA transcript^7,8^. There are currently no disease-modifying treatments for HD. Multiple attempts to target downstream pathways related to excitotoxicity, mitochondrial dysfunction, and inflammation have thus far met with little success^9^. More recent studies of pridopidine^10^, coenzyme Q10^11^, and dimebon^12^ show some benefit, although results have not always been consistent.

As an alternative to therapeutic approaches that target downstream pathways, suppression of expression of the mutant *HTT* allele has the considerable theoretical advantage of simultaneously preventing dysfunction of most or all of these pathways. Preclinical studies of *HTT*-lowering via RNA interference (RNAi), antisense oligonucleotides (ASOs), and synthetic zinc finger repressors have demonstrated the potential of this approach, with evidence of amelioration of HD pathology and increased survival in animal models^13^. These RNA-targeting methods are undergoing rapid development. Among the most salient developments, a recent phase 1/2a trial of a nonallele-specific modified ASO targeting the HD transcript demonstrated tolerability and decreased CSF levels of mHTT, a biomarker of disease activity^14^; a phase 3 trial is now underway (ClinicalTrials.gov NCT03342053). An allele-specific ASO approach is in the midst of two 1a/2b clinical trials (ClinicalTrials.gov NCT03342053 and NCT03225833). A phase 1/2 trial using adeno-associated virus (AAV)-expressed RNAi at an early stage of disease is also underway (ClinicalTrials.gov NCT04120493). Gene editing approaches are another intriguing alternative, still in the early stages of development^15^. While very exciting, these approaches face numerous challenges: off-target inhibition of other genes, immunostimulation, lack of specificity for the mutant allele, the need for broad brain distribution, and the development of clinically feasible delivery mechanisms.

A recently discovered gene antisense to *HTT* (*huntingtin antisense, HTT-AS*)^16^ represents another potential drug target. *HTT-AS* falls within the category of natural antisense transcripts, a subset of long non-coding RNAs (lncRNAs) which at least partially overlap with a gene on the opposite strand. As many as 72% of genes in mice and 40% in humans may have corresponding antisense genes^17^. These are typically expressed at much lower levels than the sense transcript, but often have critical regulatory roles. For instance, in endothelial cells, the expression of *sONE*, the antisense transcript to *eNOS*, is about 500 × lower than *eNOS*. Nonetheless, stimulation of *sONE* expression with the small molecule TSA (a histone deacytylase inhibitor) leads to an 80% decrease in *eNOS* protein expression, apparently via post-translational mechanisms^18,19^. Similar examples exist in genes relevant to neurological diseases. The expression level of a splice variant of *ASFMR1*, the gene antisense to fragile X mental retardation gene (*FMR1*), may influence the risk for development of the fragile X tremor ataxia syndrome (FXTAS)^20^. *HTT-AS* and *HTT* appear to have a similar relationship: in cell systems, overexpression of the *HTT-AS* transcript in cis resulted in a decrease in endogenous *HTT* transcript levels, while siRNA knockdown of *HTT-AS* increased *HTT* transcript levels^16^.

The manipulation of natural antisense transcripts (NATs) is of therapeutic interest, particularly through AntagoNATs, a term coined to describe modified oligonucleotides that interfere with sense-antisense interactions^21^. AntagoNATs were used to target BDNF-AS to increase BDNF transcript levels by 2–7 ×; similar effects were observed with AntagoNAT suppression of NATs of both GDNF and EPHB2. AntagoNAT-induced decrease of the transcript antisense to the SCN1A gene results in upregulation of SCNA1, with phenotypic improvement in a Dravet syndrome mouse model^22^. Knockdown of *SMN-AS1* in fibroblasts by treatment with a chemically modified oligonucleotide increased *SMN2* expression sixfold, a potential approach to the degenerative disease spinal muscular atrophy (SMA)^23^. As therapeutic agents AntagoNATs have the same advantages and disadvantages of other oligonucleotide-based approaches, as noted above.

Alternatively, small molecules that penetrate the blood–brain barrier do not require intraventricular or intrathecal infusions and avoid some of the safety concerns associated with the viral delivery typically required for shRNA-based therapeutics^24^. Screens using assays in which promoters were linked to a luciferase reporter have yielded small molecules that upregulate expression of the Notch pathway-associated transcription factor *ATOH1*^25^, suppress expression of *HAMP* (which encodes hepcidin, a regulator of iron homeostasis)^26,27^, and suppress expression of *PHOX2B*, a gene involved in neurogenesis^28^. Optimism for the use of small molecules to modify gene expression has been encouraged by the success of risdiplam, a small molecule that modifies SMN2 splicing, increasing full length expression of SMN2 to correct the loss of SMN1 in patients with spinal muscular atrophy^29^. Risdiplam improved patient outcome in multiple clinical trials (ClinicalTrials.gov NCT03779334, NCT02908685, NCT02913482, NCT03032172), and recently received FDA approval for use in SMA patients^30^. A second agent, branaplam, also acts on SMN2 splicing^31^ and is currently entering clinical trials (ClinicalTrials.gov NCT02268552).

In HD, attempts to find small molecules that decrease HTT expression have thus far not yielded agents of sufficient interest to reach the stage of clinical trial^1,32^. Recognition that mHTT itself has a widespread effect, direct and indirect, on expression of other genes^33,34^ has led to attempts to reverse these effects, including efforts to inhibit DNA methyltransferases, histone deacetylases, and histone methyltransferases^35^. Clinical progress has been limited due to pleiotropic targets and evidence of toxicity.

We developed quantitative high-throughput screening (HTS) assays for small compounds that act either to suppress activity of the *HTT* promoter or activate the *HTT-AS* promoter. The HTS was followed by a rigorous multistep validation process in which HTT protein expression and cytotoxicity was examined in 5 different disease relevant cell systems. While many compounds demonstrated apparent activity at the promotor level, none of them were both efficacious and non-toxic in all the validation assays. We review the advantages and disadvantages of this rigorous approach to a drug screen for HTT expression modulators, and comment on a few compounds that remain of interest.

## Materials and methods

### Compound libraries

All compound libraries used in this study are listed in Table 1. Identity of compounds can be found in the NCBI PubChem repository (Assay IDs: 1508621, 1508622, 1508623, 1508624).

**Table 1:** Compound libraries used in this study.

Library	Number of compounds	Details
LOPAC	1280	Known function, commercially available (Sigma)
Mechanism Interrogation PlatE (MIPE)	1912	Pharmacologically defined small molecules with relevance to cancer, neurology, infectious diseases and stem cell biology
NPC	2416	Library of all compounds which have been approved by the Food and Drug Administration, as well as a number of approved molecules from related agencies in foreign countries
Sytravon	46,361	Diversity, no biological information. A retired Pharma screening collection containing a diversity of novel small molecules, with an emphasis on medicinal chemistry-tractable scaffolds
Epigenetic	72	Epigenetic Regulators

### Cell lines and culture methods

The cell lines used in this study are listed in Table 2. Flp-In T-Rex Human Embryonic Kidney 293 (HEK293) cells were purchased from ThermoFisher Scientific, while the parental HEK293 cell line was obtained from ATCC (Manassas, VA, USA). Both HEK293 lines were grown in Dulbecco’s modified Eagle’s medium (DMEM) supplemented with 10% fetal bovine serum (FBS) and 1 × Penicillin–Streptomycin-Amphotericin B (P/S/A). GM05539 lymphoblastoid cells from a male HD patient were obtained from the Coriell Institute for Medical Research and grown in Roswell Park Memorial Institute 1640 (RPMI1640) medium supplemented with 15% FBS and 1 × P/S/A. ST*Hdh*^*Q7*^ and ST*Hdh*^*Q111*^ cells (a gift from Marcy MacDonald) were grown in DMEM supplemented with 1% FBS, 5 mM sodium pyruvate and 0.3 × P/S as previously described^36^. All cells lines were cultured in 37 °C incubators at 5% CO_2_, except for ST*Hdh* cells which were cultured at 33 °C.

**Table 2:** Cell lines used in this study.

Name	Cell type	HD/control	Long CAG repeat size
GM05538	Lymphoblastoid	HD	86
Flp-In T-Rex 293	Human embryonic kidney	Control	N/A
HEK293	Human embryonic kidney	Control	N/A
Q72 iPSCs	Induced pluripotent stem cells	HD	72
GENEA020	Human embryonic stem cells	HD	48
STHdh^Q7^	Immortalized mouse striatal neurons	Control	N/A
STHdh^Q111^	Immortalized mouse striatal neurons	HD	111

### Stem cell differentiation and compound treatment

Neural progenitor cells derived from induced pluripotent stem cells (iPSCs)^37^ were revived from day in vitro (DIV) 18 onto 6-well plates and differentiated into human cortical neurons (hCNs) until DIV25, at which point cells were seeded onto coated 24-well plates and penicillin/streptomycin was added to media. Media was topped up at DIV26 and cells underwent a 50% media change at DIV29. Cells were incubated with compound from DIV32 to DIV39, at which point lysates from each plate were split for use in two cell-based assays. For assays involving the GENEA020 human embryonic stem cells (hESCs)^38^, cells were plated onto collagen-coated plates and allowed to attach overnight prior to treatment with compound for 48 h. Lysate from one plate was split for use in three cell-based assays.

### Dual-luciferase luminescence measurement

Baseline promoter activity of stable expression cell lines was measured in 96-well white-walled clear-bottom plates plated with 10,000 cells in 100 μL growth media per well. Doxycycline was added to CMV tet-on promoter cells in a volume of 25 μL during plating. Luminescence measurements were made following 72 h of culture at 37 °C. Validation experiments were conducted in 96-well white-walled plates containing 60,000 cells per 100 μL per well cultured for 24 h. Compounds were added in a volume of 25 μL per well and incubated for 24 h at 37 °C. Treatment media was replaced with 50 μL fresh growth media prior to luminescence measurements. High-throughput screening, measuring luminescence from a luciferase reporter, was performed in 1536-well white-walled tissue-culture treated plates. Briefly, 2500 cells were plated in 4 μL of Opti-MEM per well using a Multidrop Combi Reagent Dispenser (ThermoFisher) and cultured for 24 h. The compound libraries were transferred in a volume of 23 nL per well. Cells were then grown for 24 h at 37 °C prior to luminescence measurements. In all cases, luminescence was measured using the Dual-Glo Luciferase Reporter Assay System (Promega) following the manufacturer’s instructions.

### Time-resolved fluorescence energy transfer (TR-FRET) assay for total HTT and mutant HTT protein levels

For initial TR-FRET assays, 4000 HEK293 cells or 8000 lymphoblastoid cells were plated in a 1536-well plate in 6 μL of growth media per well and cultured overnight (~ 16 h). Compounds were added to wells in 23 nL and incubated for 24 h. Cells were lysed with 2 μL of Cisbio-Lysis buffer #2 and incubated at room temperature for 2 h. Antibodies (Table 3) were added to the lysate in a volume of 2 μL and the plate was incubated at 4 °C for 23 h. Time-resolved fluorescent signal was measured at 665 and 615 nm using an Envision Multimode Plate Reader (Perkin Elmer) to determine FRET ratio for each well. For assays in HEK293 and lymphoblastoid cells, mutant and total HTT were measured. For assays in hESCs, lysate collected from compound-treated cells was used for a multiplexed mutant HTT, total HTT, and AKT. For assays in cortical neurons, lysate was used for a multiplexed mHTT and Tau assay.

**Table 3:** Antibodies used in HTRF assays.

Antibody	Role	Emission (nm)
Anti-MAB-2166-d2	Acceptor	665
Anti-2B7-Terbium	Donor	615
MW1-Alexa488	Acceptor	520
Total-AKT-Eu^3+^-Cryptate	Donor	620
Total-AKT-d2	Acceptor	665
Total-Tau-Eu^3+^-Cryptate	Donor	620
Total-Tau-d2	Acceptor	665

### Cell viability assays

For all ATP-based cell viability assays, the Cell Titer Glo kit (Promega) was used according to manufacturer’s instructions. For total nuclear stain in hESCs, cells were fixed and permeabilized followed by Hoechst 33342 staining. Cells were imaged using the InCell2200 widefield automated microscope (GE Healthcare) and data analyzed using the InCell Developer software.

The ST*Hdh*^*Q111*^ 1536-well viability assay was performed as previously described^39^. Briefly, cells were plated in black-wall, clear-bottom 1536-well cyclic olefin polymer-type imaging plates (Edition Eight; Whitefish, MT) at 1200 cells per well in 5 μL volume using a Multidrop Combi Reagent Dispenser (ThermoFisher). Cells were incubated for 16 h in an incubator at 33 °C and 5% CO_2_. 46 nL of compounds were transferred using a pin-transfer tool and plates were returned to 33 °C for 2 h. Cells were then shifted to 37 °C and 5% CO_2_ for 24 h prior to staining and imaging. Hoechst 33342 and propidium iodide (PI) were prepared in phosphate buffer solution (PBS) and 1 μL was added to each well; final concentration was 4 μg/mL Hoechst 33342 and 5 μg/mL PI. Plates were incubated at room temperature for 30 min prior to imaging on an InCell2200 microscope using a 10 × 0.45 NA air objective and standard DAPI (390/18 ×, 432/48 m) and Cy3 (542/27 ×, 587/45 m) filter sets. One field of view per well, encompassing the entire well, was chosen for imaging and percent PI positive cells were determined using Columbus Image Data Analysis System (PerkinElmer) to identify Hoechst nuclei and PI positive nuclei on a cell-by-cell basis. A mean PI intensity of more than 3 standard deviations above the background mean was classified as PI positive. Data were normalized to controls on a per-plate basis (Q7 + vehicle = 100% protection, and Q111 + vehicle = 0% protection).

### Data analysis

Concentration–response curves (CRCs) were generated using NCATS software (https://tripod.nih.gov/curvefit/) and analyzed using previously described methods^40^. Curves are classified based on quality of fit to the data, the magnitude of response, and the number of asymptotes to the calculated curve. A positive CRC would indicate a positive correlation of activity with dose concentration, while a negative CRC would indicate a negative correlation. Structural clustering of active compounds was performed using Leadscope Hosted Client (Leadscope Inc., Columbus, OH). The EC50 values of compounds in the confirmation and follow-up experiments were calculated from the dose–response curves by nonlinear regression analysis using Prism software (GraphPad Software, San Diego, CA).

## Results

### Development of stable reporter cell lines to measure *HTT* and *HTT-AS* promoter activity

**Figure 1:**
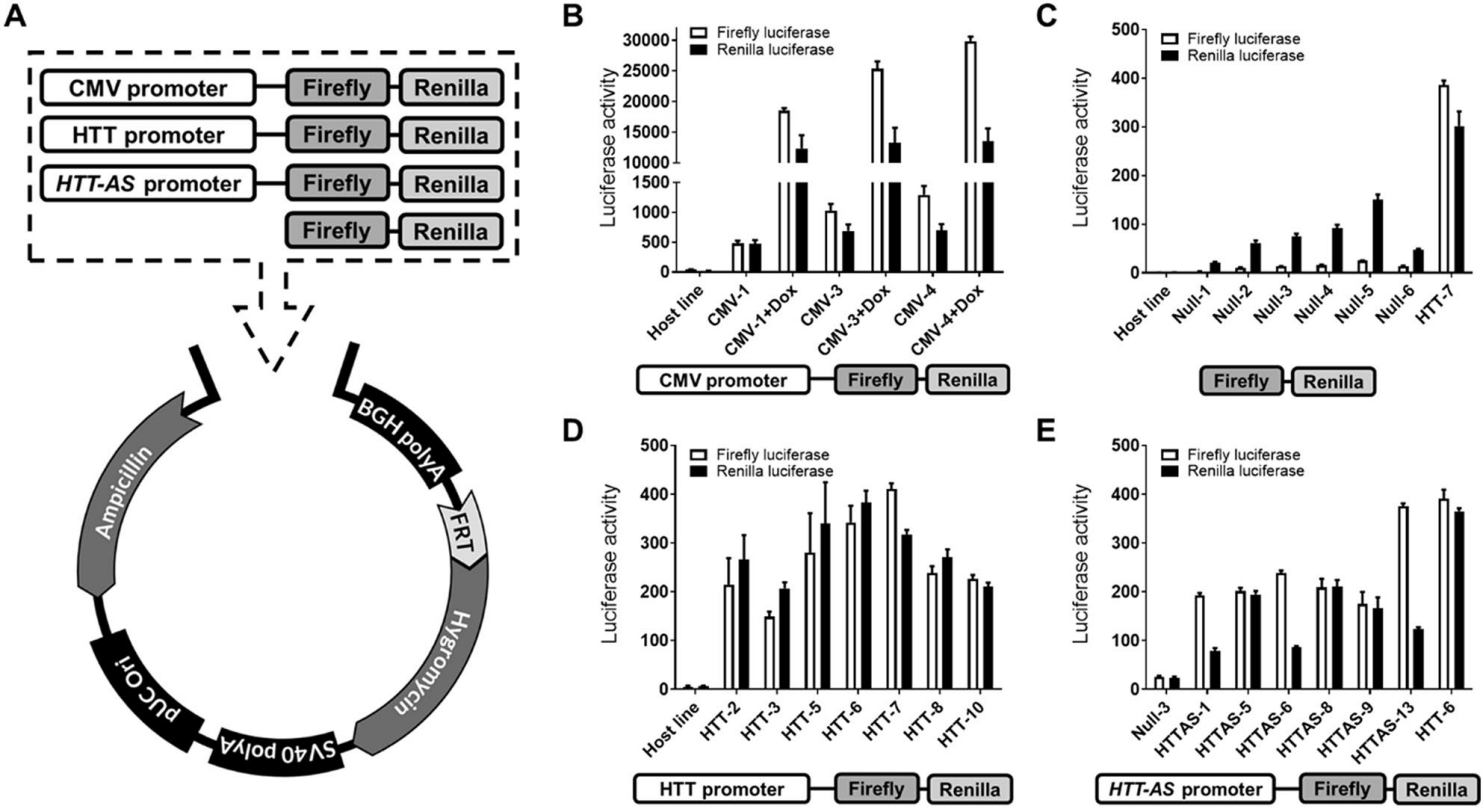
Strategy for generation of stable cell lines. (**A**) Constructs containing a promoter linked to two luciferase reporters were cloned into a pcDNA5/FRT backbone. Plasmids were transfected into HEK293 host cells with an integrated Flp recombination site. (**B**–**E**) Baseline luciferase activity was assayed in clones expressing firefly linked to (**B**) CMV promoter, with or without doxycycline (Dox) treatment (n = 3), (**C**) no promoter (“Null”; n = 4), (**D**) *HTT* promoter (n = 4), and (**E**) *HTT-AS* promoter (n = 4). Raw luminescence signal values are shown (luciferase activity); error bars are ± SD.

In order to identify small molecules that alter *HTT* or *HTT-AS* expression, we developed a reporter-based screen to assess the effects of small molecules on their respective promoters. We transfected Flp-In T-Rex HEK293 cells with a dual-reporter gene plasmid linked with the *HTT* (1.7 kb) or *HTT-AS* (1.5 kb) promoter. The *HTT* promoter sequence included 1736 bp upstream of the transcription initiation site (chr4:3073089–3074829 human genome 38; hg38)^41^. The *HTT-AS* promoter sequence (1503 bp upstream of the transcription initiation site; chr4:3074930–3076433 hg38) was chosen based on previously published reporter experiments^16^. Additional control plasmids were also constructed, including no promoter (“Null” control), or a CMV tet-on inducible promoter (Fig. 1A). We performed stable monoclonal selection and analyzed a series of monoclones. HTT-2 and HTT-AS-8 clones were selected based on results showing uniform baseline firefly and renilla luciferase activities (Fig. 1B–E), normal cell morphology, lack of effect of DMSO (1%) on growth and reporter expression, high proliferation rates, and no loss of transgene through at least P10.

### Primary screen: screening for compounds which activate the *HTT-AS *promoter or inhibit the *HTT* promoter using a luciferase reporter system

52,041 compounds from five compound libraries (Table 1) were tested for their effect on the *HTT* and *HTT-AS* promoter-expressing cell lines. Compounds were screened at four concentrations (57, 11, 2.3, and 0.46 μM). Renilla reporter results were excluded due to low signal-to-noise ratio (Supplement Figure 1). The primary screen yielded 898 compounds (hit rate = 1.73%) which had active concentration response curves (CRCs) indicating increased expression of *HTT-AS* (curve class 1/2/3) or decreased expression of *HTT* (curve class − 1/− 2/− 3). Of the 898 total actives, 809 inhibited the *HTT* promoter, 92 activated the *HTT-AS* promoter, and 3 compounds had both effects. The 898 compounds from the primary screening could also be subdivided into the following five activity profiles:

1. Decrease in *HTT* and *HTT-AS* promoter activity (687); potential general transcriptional inhibitors.
2. Increase in *HTT* and *HTT-AS* promoter activity (84); potential general transcriptional activators.
3. Decrease in *HTT* promoter activity, increase in *HTT-AS* activity (3); desirable profile (both).
4. Decrease in *HTT* promoter activity, no change in *HTT-AS* activity (119); desirable profile (*HTT*).
5. No change in *HTT* promoter activity, increase in *HTT-AS* activity (5); desirable profile (*HTT-AS*).

### Confirmation screen: identification of reproducible hits from dual-reporter screen

**Figure 2:**
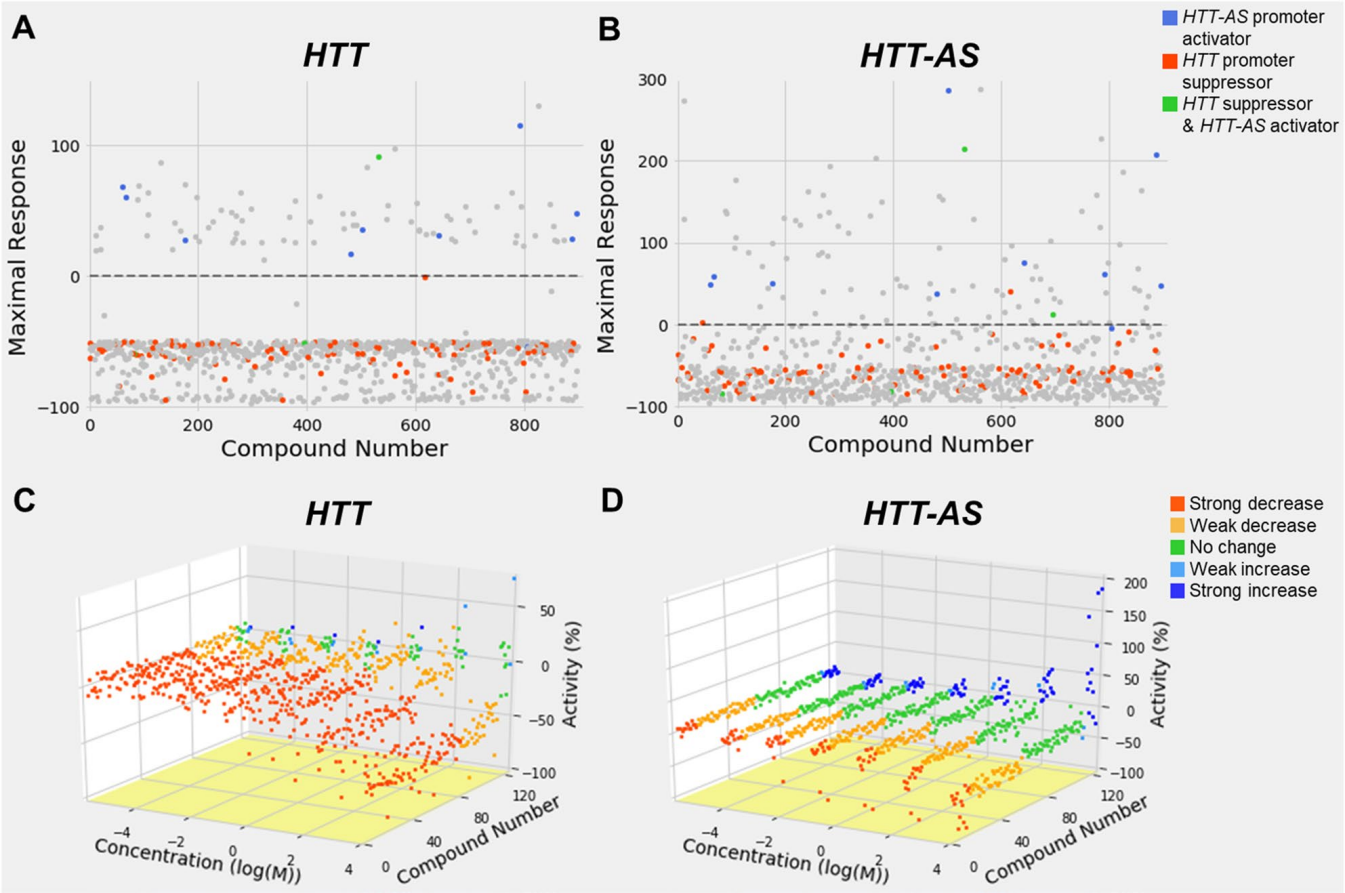
Replication of compound *HTT* and *HTT-AS* promoter activity. *HTT* or *HTT-AS* stable reporter cell lines were treated with 898 compounds identified in the HTS to replicate the initial findings. (**A**,**B**) Maximal response of luciferase activity at the highest dosage of compound treatment (57 μM) in (**A**) *HTT-* and (**B**) *HTT-AS*-promoter reporter cells. Hits were determined based on CRC values and are indicated in red (*HTT* downregulators; *HTT* CRC = − 1/− 2/− 3), blue (*HTT-AS* upregulators; *HTT-AS* CRC = 1/2/3), and green (*HTT* downregulators AND *HTT-AS* upregulators). The remaining compounds with unfavorable or inactive CRCs are indicated in gray. One outlier (449%) from (**A**) and 3 outliers (843%, 458%, 403%) from (**B**) are not shown. (**C**,**D**) Waterfall plots of luciferase activity in (**C**) *HTT* and (**D**) *HTT-AS* reporter cells in response to treatment by 123 compounds chosen for further study. Red: strongly inactivating compounds (CRC = − 1.1/− 1.2/− 2.1/− 2.2). Orange: weakly inactivating compounds (CRC = − 1.3/− 1.4/− 2.3/− 2.4/− 3). Green: inactive compounds (CRC = 4/5). Cyan: weakly activating compounds (CRC = 1.3/1.4/2.3/2.4/3). Blue: strongly activating compounds (CRC = 1.1/1.2/2.1/2.2).

898 compounds identified in the initial promoter screen were reevaluated at 7 concentration (57 μM top concentration, 1:3 titration) to confirm their dose–response activity based on CRCs (1/2/3 for *HTT-AS* or − 1/− 2/− 3 for *HTT*) (Fig. 2). 739/809 *HTT* lowering compounds (91%) and 52/92 (57%) *HTT-AS* activating compounds confirmed the validation screen, for a composite confirmation rate of 88% (791/898). Compounds that were active in the confirmation screen were further triaged by structural clustering, elimination of compounds with promiscuous and undesirable functionalities, and removal of compounds which showed an increase in *HTT* promoter activity as such compounds were unlikely to prove of clinical interest. 123 compounds met these criteria and were selected for further study. Of this group, 112 decreased *HTT* promoter activity and 15 increased *HTT-AS* activity, with four compounds both decreasing *HTT* and increasing *HTT-AS* promoter activity.

### Secondary screen: effect on huntingtin protein in HEK293 and HD patient lymphoblast cell

In order to ascertain huntingtin protein-lowering capacity, a FRET-based assay was used to measure the dose–response effect of the 123 selected compounds on huntingtin protein levels. Compounds were screened at 10 concentration dose response in both HEK293 cells (38 μM with 1:3 dilutions) and a lymphoblast cell line from an HD patient (long allele = CAG_82_; 17 μM with 1:3 dilutions). HD patient-derived lymphoblasts were selected for use in this study as they have been successfully used to identify novel aspects of HD pathophysiology^42–44^, as well as evaluate the effectiveness of potential therapeutic agents^45,46^. An ATP-measuring assay was performed in parallel using the same concentration range to determine compound toxicity.

**Figure 3:**
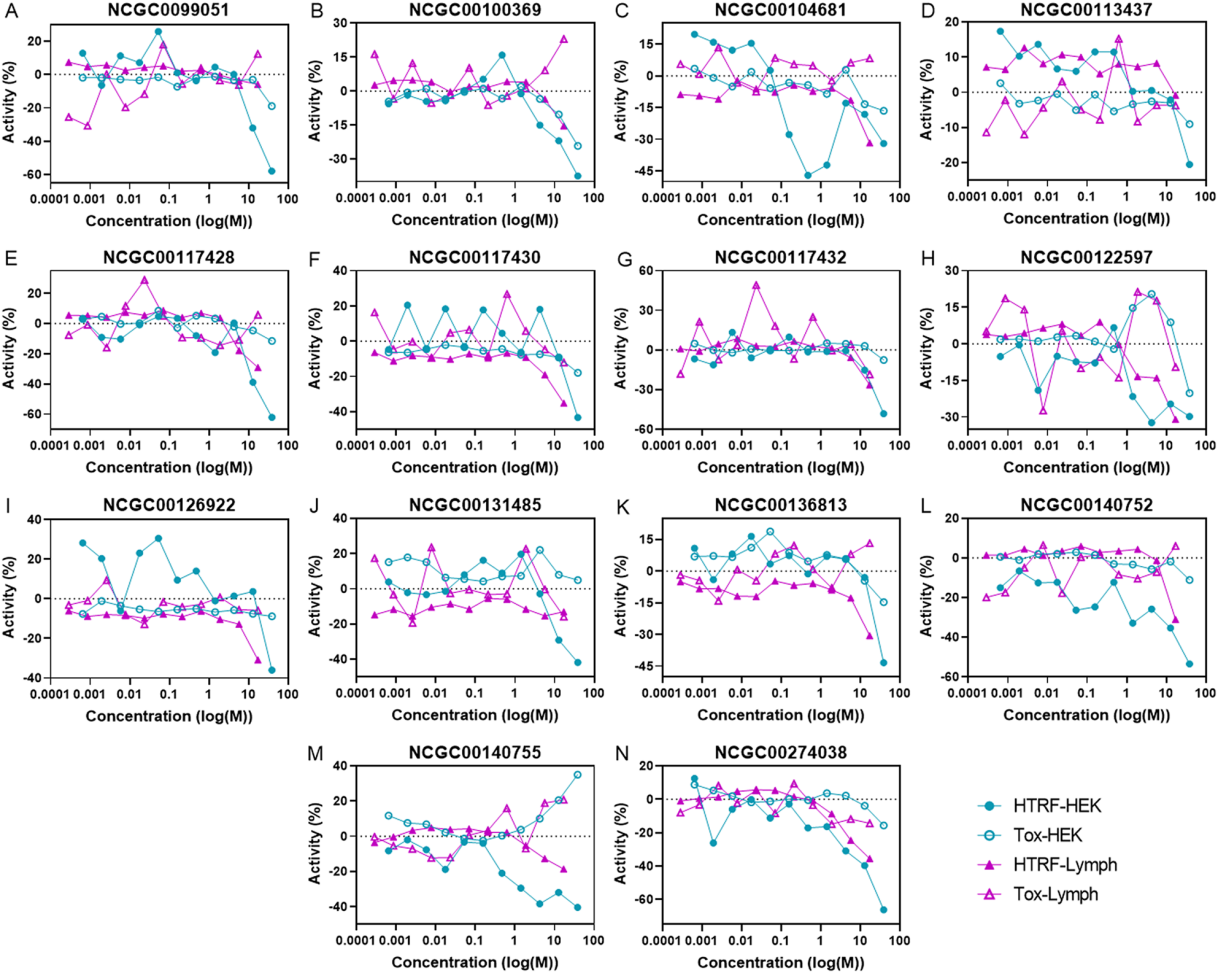
Huntingtin protein-lowering and cytotoxicity assays in HEK293 cells and HD lymphoblasts of 123 compounds that altered *HTT* or *HTT-AS* promoter activity. 14 compounds (A–N) out of 123 tested lowered huntingtin protein in either HEK 293 cells (HTRF-HEK) or HD Lymphoblasts (HTRF-Lymph) based on an HTRF assay and were non-toxic according to ATP-based cytotoxicity assays (Tox-HEK; Tox-Lymph). Each compound was screened at 10 concentrations (HEK293 screen, highest concentration = 38 μM, with 1:3 dilutions; Lymphoblast screen, highest concentration = 17 μM with 1:3 dilutions; single well per concentration). Compounds were chosen based on active CRCs for huntingtin lowering (− 1/− 2/− 3) and inactive CRCs for cytotoxicity (≥ 4). Values are normalized to measurements in untreated HEK293 and HD lymphoblast cells.

A total of 17 compounds strongly lowered huntingtin protein levels as measured by the Homogeneous Time-Resolved Fluorescence (HTRF) assay, and evaluated by CRCs (− 1.1/− 1.2/− 2.1/− 2.2): 10 were active in HEK293 cells, 4 in HD lymphoblasts, and 3 in both cell lines. After eliminating toxic compounds, 14 were classified as active (Table 4; Fig. 3); 10 in HEK293 cells only, 6 in HD lymphoblasts only, and 2 compounds in both cell lines. Of the two compounds that lowered huntingtin protein in both cell types, one (NCGC00274038) was a hit derived from the *HTT-AS* screen, and one (NCGC00104681) from the *HTT* screen.

**Table 4:** Summary of compounds that showed huntingtin protein-lowering activity in the HEK293 and HD lymphoblast assays.

Compound name	ID	Promoter screens (AC_50_; μM)^†^		Protein-lowering screens (AC_50_; μM)			Neuron and stem cell-based screens (AC_50_; μM)			
		*HTT*	*HTT-AS*	HEK293 (HTT)	HD Lymph (HTT)		HD hESC (mHTT)	HD hCN (mHTT)	mStN^††^	
									AC_50_	Eval
NCGC00099051	A	19.01	Null	11.87	Null		30.0	18.4	15.34	Toxic
NCGC00100369	B	37.93	3.01	11.87	Null		30.0	7	43.24	Protective
NCGC00104681	C	21.33	Null	0.07	11.25		30.0	Null	N/T	N/T
NCGC00113437	D	15.10	Null	14.95	Null		30.0	Null	19.31	Protective
NCGC00117428	E	3.79	Null	11.87	Null		N/T	N/T	N/T	N/T
NCGC00117430	F	10.69	Null	Null	7.97		28.18	7.1	N/T	N/T
NCGC00117432	G	21.33	Null	21.33	Null		30.0	6.2	N/T	N/T
NCGC00122597	H	1.69	Null	Null	4.48		1.91	0.893	7.69	Protective
NCGC00126922	I	26.85	Null	Null	11.25		30.0	Null	N/T	N/T
NCGC00131485	J	4.78	Null	4.78	Null		Null	14.8	Null	Inactive
NCGC00136813	K	37.93	1.20	Null	11.25		28.18	Null	48.52	Protective
NCGC00140752	L	665	Null	16.77	Null		30.0	Null	10.86	Protective
NCGC00140755	M	620	Null	0.75	Null		N/T	N/T	N/T	N/T
NCGC00274038	N	Null	23.93	9.43	3.99		3.98	Null	1.72	Toxic

*N/T* = *Not tested.*

^†^AC_50_ indicates luminescent suppression (*HTT*) or induction (*HTT-AS*).

^††^AC_50_ indicates cytoprotection or cytotoxicity.

### Tertiary screen: huntingtin protein lowering in stem cell and neuronal HD lines

12 out of the 14 compounds active compounds in the HTRF assay above were further tested in human embryonic stem cells (hESCs) from an HD patient (Charles River, long allele = CAG_48_) and cortical neurons differentiated from an HD induced pluripotent stem cell (iPSC) line (Evotec, long allele = CAG_72_). Two compounds were excluded due to hazardous material transportation restrictions or limited supplies.

**Figure 4:**
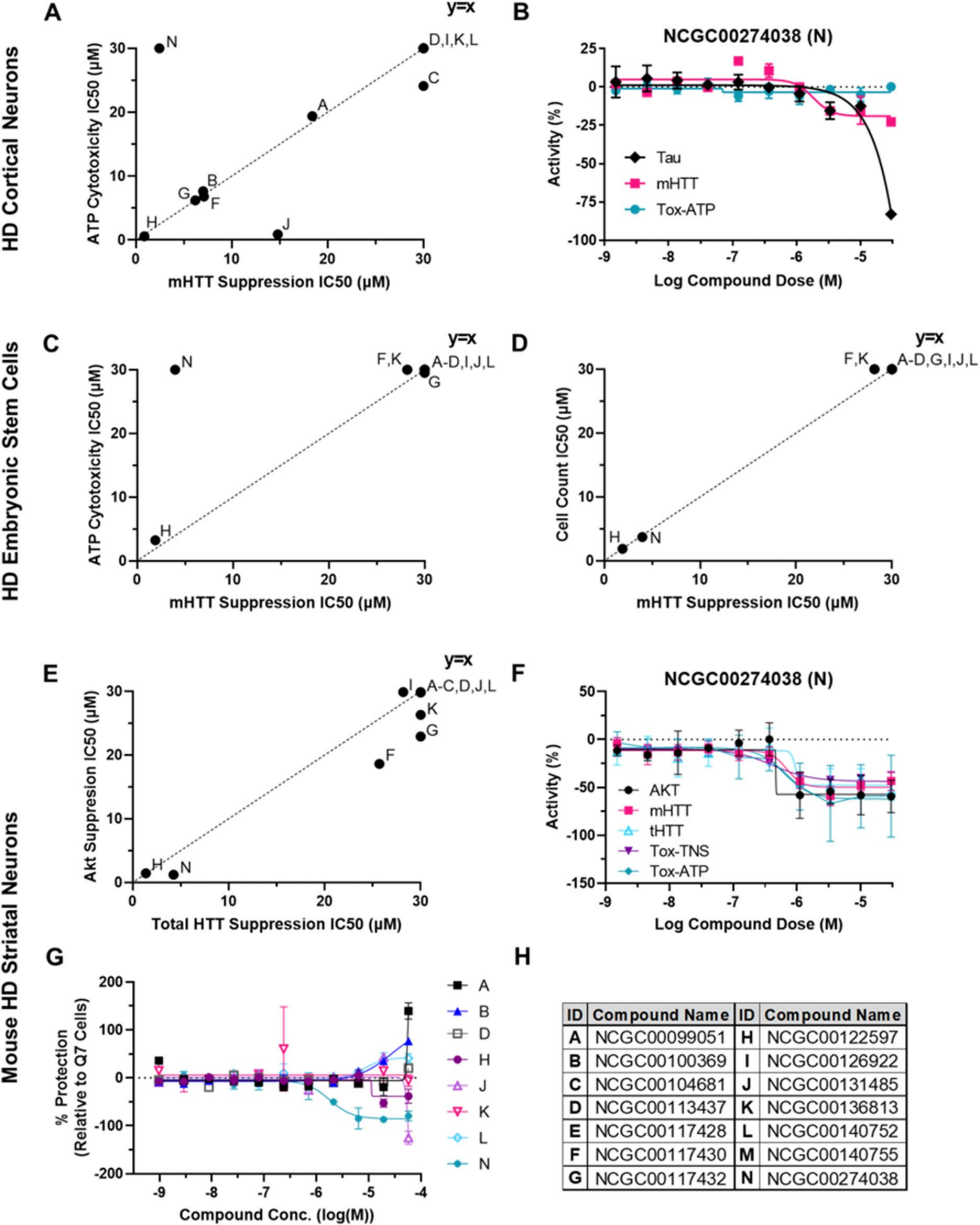
Tertiary screening for huntingtin-lowering and toxicity in HD neuronal and stem cell lines. 12 compounds were tested in (**A**,**B**) HD cortical neurons and (**C**–**F**) HD embryonic stem cells, and 8 compounds in (**G**) HD mouse striatal cells. HTRF assays were used to measure mHTT, total HTT, Akt, and Tau concentrations at each dosage. Cytotoxicity was determined by total nuclear cell count (Tox-TNS) or ATP-measurement assays (Tox-ATP). (**A**) mHTT suppression IC_50_ is plotted against ATP-based cytotoxicity. One compound (NCGC00274038; N) out of 12 shows ability to lower mHTT suppression with a window over cytotoxicity. (**B**) Dose–response curve of NCGC00274038 Tau protein suppression, mutant HTT (mHTT) protein suppression and ATP-based cytotoxicity (Tox-ATP) in HD cortical neurons. Values are normalized to measurements in untreated cells. Error bars are ± SD; n = 2. (**C**) mHTT suppression IC_50_ is plotted against ATP cytotoxicity IC_50_. NCGC00274038 suppresses mHTT with a window over ATP-based cytotoxicity in HD hESCs. (**D**) mHTT suppression IC_50_ is plotted against cell count-based cytotoxicity IC_50_. No compounds show ability to suppress mHTT with a window over cell count-based cytotoxicity. (**E**) Total HTT suppression IC_50_ is plotted against cell count-based cytotoxicity IC_50_. No compounds show specific suppression of total HTT over Akt. (**F**) Dose–response curve of protein suppression (Tau, mutant HTT, total HTT) as well as ATP-based (Tox-ATP) and cell count-based (Tox-TNS) cytotoxicity in HD hESCs in response to NCGC00274038 treatment. Values are normalized to measurements in untreated HD hESCs. Error bars are ± SD; n = 4. (**G**) Dose response curves of 8 compounds tested for protective activity in ST*Hdh*^*Q111*^ model. Proportion of live cells detected by PI (negative) was measured relative to total cells detected by Hoechst 33342 (% Protection), normalized to measurements in untreated ST*Hdh*^*Q7*^ cells. Error bars are ± SD; n = 2. (**H**) Letters in IC_50_ plots indicate compounds IDs, also shown in Table 4.

Cortical neurons were treated with compound for seven days at 10 doses (30 μM with 1:3 dilutions). A FRET-based assay was used to assess the capacity of each compound to lower mutant huntingtin, with an ATP-based cell viability assay run in parallel. One compound (NCGC00274038) exhibited some lowering of mHTT at concentrations that did not alter ATP levels (Fig. 4A,B). However, it also lowered Tau as assayed by FRET, indicating a lack of specificity. HD hESCs were treated with compound at 10 doses (30 μM with 1:3 dilutions). Both mutant and total huntingtin were measured, as well as protein kinase B (Akt) to assess specificity of action. Cell viability was measured both by an ATP-based assay and by total cell count based on Hoechst staining. One compound (NCGC00274038) showed measurable huntingtin-lowering activity relative to ATP-based cell viability (Fig. 4C–F). However, this compound had equipotent effects on huntingtin-lowering cell viability with toxicity measured by cell count. Consistent with this effect and with results in cortical neurons, Akt suppression also paralleled *HTT*-lowering, indicating non-specific action by the compound.

8 of the 14 hits derived from HEK293 and HD lymphoblast studies were also tested for their cytoprotective capacity in ST*Hdh*^*Q111*^ mouse striatal cells. Striatal neurons were treated with compound for 24 h at 11 doses (30 μM with 1:3 dilutions) while under stress conditions (low temperature, low serum). Cells were stained with Hoechst to label all nuclei and propidium iodide (PI) to detect dead cells. Percentage of PI positive neurons in compound-treated cells relative to untreated Q7 neurons was used to measure cytoprotection. Two compounds (NCGC00100369, NCGC00113437) showed strong cytoprotective effects (Fig. 4G), three compounds showed mild cytoprotective effects (NCGC00122597, NCGC00136813, NCGC00140752), two compounds were cytotoxic (NCGC00099051, NCGC00274038), and one compound was inactive (NCGC00131485).

## Discussion

**Figure 5:**
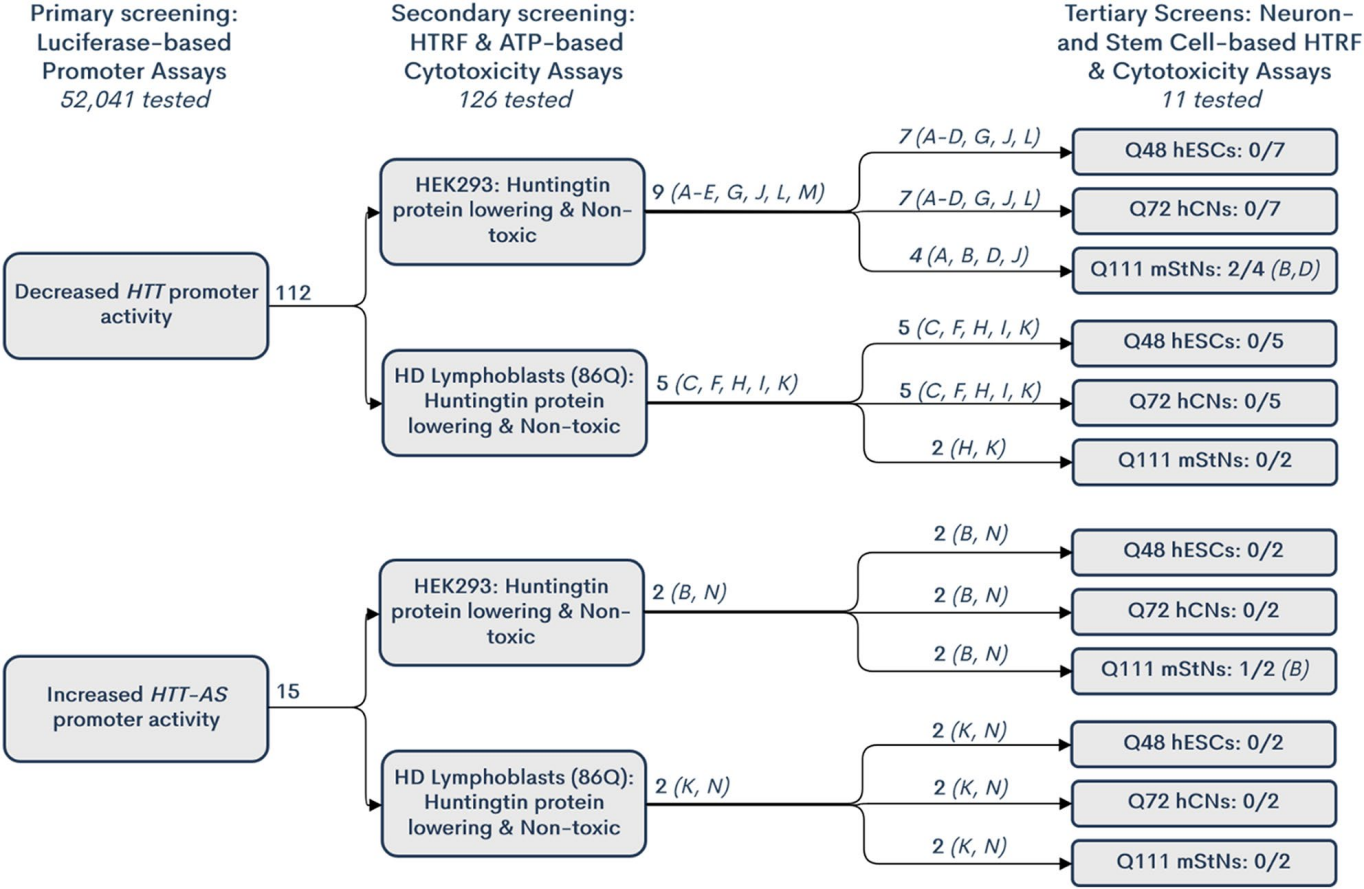
Summary of high throughput screening for *HTT* and *HTT-AS* promoter modifiers. 52,041 compounds were tested in the primary screen, with a total of 123 hits (112 hits in the *HTT* promoter assay and 15 in the *HTT-AS* promoter assay, with 4 compounds active in both assays. Of these 123 compounds, 14 were identified as huntingtin-lowering and non-toxic in the secondary screen. 10 were found to lower huntingtin protein in HEK293 cells and 6 in HD-patient derived lymphoblasts, with 2 active in both cell types. 12 of these compounds were tested in HD human embryonic stem cells (hESCs) and HD cortical neurons (hCNs), and 8 in immortalized HD mouse striatal neurons (mStNs). Compound IDs (Table 4) in chart indicate which compounds moved forward to each tertiary screen. 2 compounds (NCGC00100369; B) and (NCGC00113437; D) were identified as active in the mStNs, but not in hESCs or hCNs.

We developed a high-throughput luciferase reporter assay to screen for compounds that act on the *HTT* or *HTT-AS* promoter, using HEK293 cells expressing the *HTT* or *HTT-AS* promoter linked to a luciferase reporter. This assay is 1536-well compatible, enabling us to screen a diverse collection of ~ 50,000 compounds. We identified 739 compounds that reproducibly decreased *HTT* promoter-driven luciferase activity, and 52 that increased *HTT-AS* promoter-driven luciferase activity. Validation, based on the capacity of hits to lower huntingtin protein at non-toxic concentrations, was performed in two tiers, first, using HEK293 cells and lymphoblasts from HD patients; and second, using HD patient ESCs and iPSCs, and primary neurons from an HD mouse model. None of the small molecules identified in the initial screen were efficacious at non-toxic doses in all tests of validity (Fig. 5).

The discrepancy of results between HEK cells, lymphoblasts, and neuronal and stem cell lines may in part derive from differences in the mechanisms that regulate HTT expression. While huntingtin is ubiquitously expressed, expression levels are presumably under at least some cell-type specific regulation, as indirectly demonstrated by varying levels of HTT expression in different tissues (e.g., the expression of normal HTT is 5 × higher in transformed lymphocytes than in caudate). Other small molecules detected by moderate- or high-throughput screenings as potential HD therapeutic agents have also acted differently on specific cell types, and different assays may yield different results in the same cell-type^47,48^. The mechanisms of *HTT* transcriptional regulation, and how this regulation differs by cell type, is poorly understood^49^. Our strategy of testing efficacy, specificity, and toxicity in non-neuronal cells first was intended to reduce false positives while increasing the efficiency and decreasing the cost of the screen. The trade-off is a likely increase in false negatives. Other high-throughput screenings for small molecule therapeutics for HD have faced similar obstacles^50,51^.

Our approach emphasized cultured neurons as a final step in validation and test of toxicity. However, cultured neurons are more vulnerable to toxicity than either neurons in vivo or in co-culture with astrocytes^52–54^, increasing the likelihood that potential compounds of interest will be screened-out based on toxicity^55^. Use of co-culture systems, previously developed for investigation of HD pathogenesis^56^, or 3D cultures^57^, will likely decrease this source of false negatives. In addition, our results reflect the methodology used for evaluation of toxicity. ATP-based toxicity screening was used to assess toxicity in HEK293 cells, HD lymphoblasts, and induced cortical neurons, while both ATP and cell count-based methods were used to assess toxicity in HD hESCs. While cell counting methods avoid the issues of cell growth that can confound assays based on ATP production, the advantage comes with the cost of scalability.

One compound, NCGC00274038, a TGBβ-activated kinase (TAK1) inhibitor, lowered HTT and was not toxic by ATP measures in induced neurons, though it was toxic when assayed by cell count in hESCs and in a mouse striatal-derived cell line. We excluded this compound from further consideration based on our a priori analytic plan and evidence that direct cell counting is superior to ATP-based assays, recognizing that this decision is quite conservative. However, this does not necessarily exclude this compound or related compounds from further investigation. Previous studies have shown TAK1 inhibitors to have neuroprotective properties^58,59^. TAK1 is a positive regulator of MAPK signaling, and dysregulation of this pathway has been implicated in HD^60^ and other neurodegenerative diseases^61^. Two recent studies have identified MAPK-related kinases as a positive regulators of mHTT protein levels, potentially via increased stability of *HTT* mRNA^62,63^. NCGC00274038 may therefore reduce *HTT* expression via suppression of MAPK signaling; its detection in our screen for compounds acting on the HTT promoter may have been artifactual, from an unrelated or indirect activity, or due to a stabilizing effect on luciferase mRNA. Other TAK1 inhibitors without the toxicity we detected in NGC00274038 may therefore merit investigation in HD, though likely independent of an effect on the HTT promoter.

This study included a screen for small molecules that upregulated *HTT-AS*, guided by the rationale that, as found in sense-antisense pairs at other loci, *HTT-AS* appears to suppress *HTT*. This screen had the advantage of minimizing detection of compounds on the basis of nonspecific toxicity. We focused on the *HTT-AS* promoter that drives transcription of *HTT-AS*-v1, which includes exon 1 with the CUG repeat. One challenge is that over-induction of this transcript may be toxic via the toxicity of expanded CUG repeats^64,65^. More generally, the regulation of expression at bidirectional loci is complex, and it is possible that small molecule manipulation of the antisense promoter is insufficient to elicit the response detected in model systems^16^. We have recently detected a second promoter that appears to drive expression of *HTT-AS*-v2; this transcript does not incorporate the *HTT-AS* exon 1, which contains the repeat, but does incorporate *HTT-AS* exon 2, which is precisely located antisense to the *HTT* promoter region. While the extent to which activating this second promoter can lead to suppression of HTT remains unclear, it may prove a more amenable target for manipulation than the promoter targeted in this study.

Our assay was designed to detect any potential activator of the *HTT* or *HTT-AS* promoter, in part based on limited information on more specific regulators of *HTT* or *HTT-AS* promoter function. If detected, such factors might provide a more focused therapeutic target. Work outside of HD suggests that other targets with the potential of lowering HTT expression might include promoter specific G-Quadruplexes^66^, the point of interaction between a transcription factor and Pol II^67^, promoter-regulating lncRNAs^68^, or translational machinery^69^.

We conclude that reducing m*HTT* expression remains an important goal in the field. Antisense and RNAi strategies look promising, but a small molecule approach may avoid some of the obstacles facing these strategies or could lead to an adjuvant therapy. Our results suggest that identifying more specific targets may facilitate this approach.

## Supplementary Information

Supplementary Figure S1.

## Data Availability

The datasets generated during the current study are available in the NCBI PubChem repository with the following Assay IDs: 1508621, 1508622, 1508623, 1508624. Note, classification of active versus inactive compounds using the PubChem Activity Score is not identical to the criterion described herein.
