# Supplementary material for: A high-throughput screening to identify small molecules that suppress huntingtin promoter activity or activate huntingtin-antisense promoter activity
Source: Sci Rep. 2021 Mar 17;11:6157. doi: 10.1038/s41598-021-85279-2 (PMC7969751; doi:10.1038/s41598-021-85279-2)
Supplement: Supplementary file 1 — Supplementary Figure S1. [file 41598_2021_85279_MOESM1_ESM.pdf]

A high-throughput screening to identify small molecules that suppress *huntingtin* promoter activity or activate *huntingtin-antisense* promoter activity

Houda G Khaled[1,2], Hongxuan Feng[1,#], Xin Hu[4,#], Xin Sun[1,3,#], Wang Zheng[1,5,#], Pan P. Li[1], Dobrila D. Rudnicki[1], Wenjuan Ye[4], Yu-Chi Chen[4], Noel Southall[4], Juan Marugan[4], Christopher A. Ross[1,6,7] Marc Ferrer[4], Mark J. Henderson[4], Russell L. Margolis[1,7,*]

[1]Laboratory of Genetic Neurobiology, Division of Neurobiology, Department of Psychiatry, Johns Hopkins University School of Medicine, Baltimore, MD

[2]Current address: Center for Neural Science, New York University, New York, NY

[3]Current address: Jacobio Pharmaceuticals Ltd., Beijing, China

[4]National Center for Advancing Translational Sciences, NIH, Rockville, MD

[5]Current Address: Center for Cancer and Immunology Research, Children's National Medical Center, Washington, DC 20010, USA.

[6]Departments of Neuroscience and Pharmacology, Johns Hopkins University School of Medicine, Baltimore, MD

[7]Department of Neurology, Johns Hopkins University School of Medicine, Baltimore, MD

#These authors contributed equally.

*Corresponding author: Russell L. Margolis, M.D., Laboratory of Genetic Neurobiology, Division of Neurobiology, Department of Psychiatry, Johns Hopkins University School of Medicine, CMSC 8-121, 600 N. Wolfe Street, Baltimore, MD, 21287 email: rmargoli@jhmi.edu; fax: 443-927-7965, phone: (office) 410-614-4262 (cell) 410-227-366

Supplemental Figure 1: Validation of high throughput promoter screening. (A-D) Results from the initial high throughput promoter screen (FF-HTS, Ren-HTS) were validated in a small-scale assay of 14 compounds (FF-Val, Ren-Val), measuring firefly (FF) and renilla (Ren) luminescence activity relative to untreated cells (% Activity). Examples shown include: (A) compound NCGC00110055 (*HTT-AS*), in which both firefly (FF-HTS, FF-Val) and renilla (Ren-HTS, Ren-Val) were reproducible, (B) NCGC00138290 (*HTT-AS*) in which only firefly results were reproducible, and (C) NCGC00104476 (*HTT*), in which neither reporter results were reproducible. (D) Summary of validation results on 14 selected compounds. Reproducibility of firefly reporter results is greater than those of renilla, which were equivalent to chance. Error bars are shown for validation experiments (n=5), representing ± SD. (E) Reproducibility of initial and confirmation high throughput screenings of promoter activity. Compounds were considered potential downregulators of *HTT* promoter activity if they received CRCs of -1/-2/-3, and potential upregulators of *HTT-AS* promoter with CRCs of 1/2/3. Reproducible negatives showed undesirable CRCs for both assays. Discrepant compounds results differed between the initial and the confirmation assay.

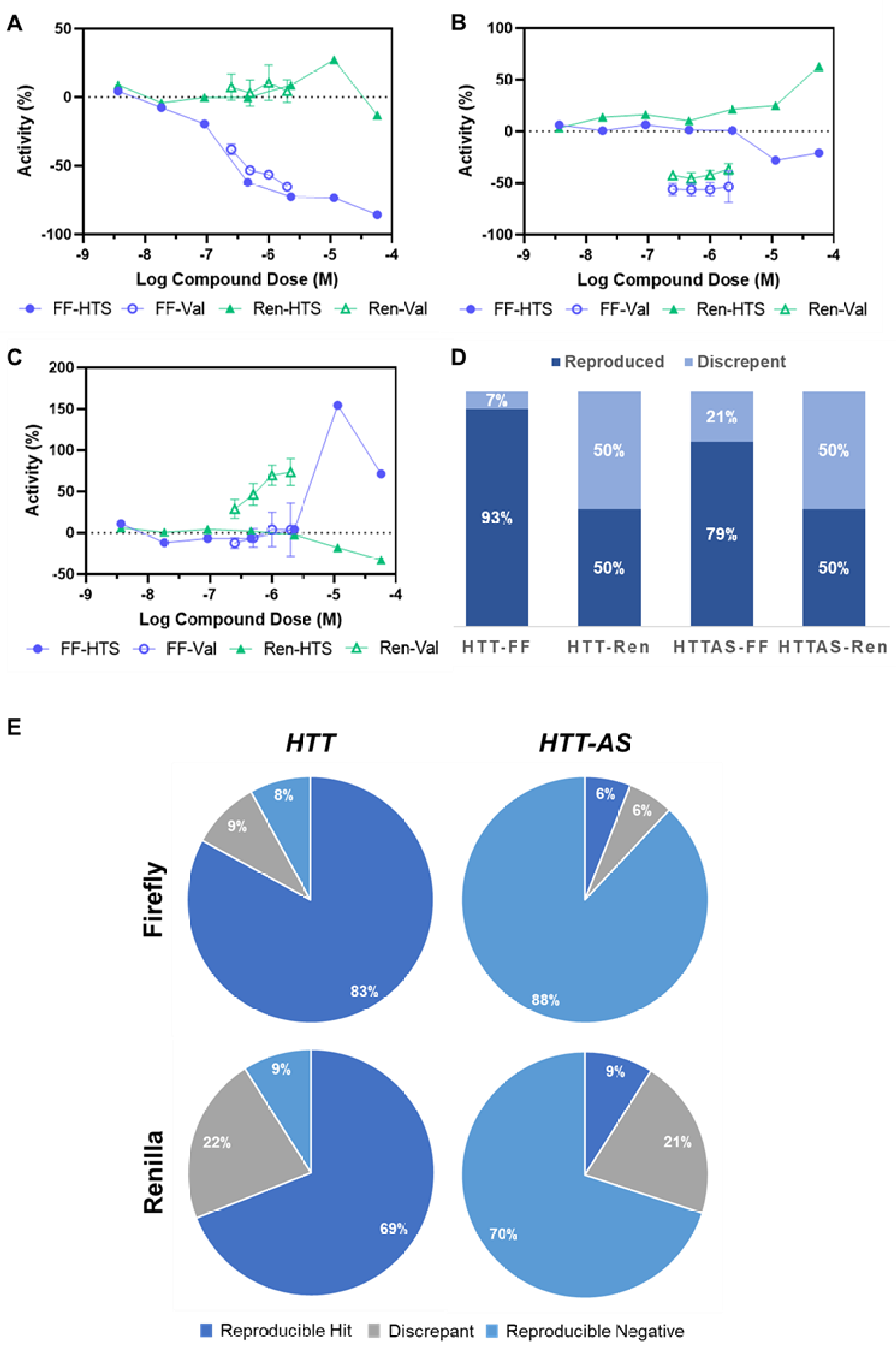
A
B
C
D
E
Activity (%)
Log Compound Dose (M)
FF-HTS
FF-Val
Ren-HTS
Ren-Val
Reproduced
Discrepent
7%
93%
50%
50%
21%
79%
50%
50%
HTT-FF
HTT-Ren
HTTAS-FF
HTTAS-Ren
HTT
HTT-AS
Firefly
Renilla
8%
9%
83%
6%
6%
88%
9%
22%
69%
9%
21%
70%
Reproducible Hit
Discrepant
Reproducible Negative
